# Structural Basis for Differences in Dynamics Induced by Leu Versus Ile Residues in the CD Loop of Kir Channels

**DOI:** 10.1007/s12035-015-9466-x

**Published:** 2015-10-31

**Authors:** Shouqin Lü, Hailong An, Hailin Zhang, Mian Long

**Affiliations:** 1 Center of Biomechanics and Bioengineering, Institute of Mechanics, Chinese Academy of Sciences, Beijing, 100190 China; 2 Key Laboratory of Microgravity (National Microgravity Laboratory), Institute of Mechanics, Chinese Academy of Sciences, Beijing, 100190 China; 3 Beijing Key Laboratory of Engineered Construction and Mechanobiology, Institute of Mechanics, Chinese Academy of Sciences, Beijing, 100190 China; 4 Key Laboratory of Molecular Biophysics, Hebei Province, Institute of Biophysics, School of Sciences, Hebei University of Technology, Tianjin, 300401 China; 5 Key Laboratory of Neural and Vascular Biology, Ministry of Education, The Key Laboratory of Pharmacology and Toxicology for New Drug, Hebei Province, Department of Pharmacology, Hebei Medical University, Shijiazhuang, 050017 China

**Keywords:** Inward-rectifier potassium channel, Molecular dynamics simulations, Conformational change, Patch clamp, Structure-function relationship

## Abstract

The effect of the conserved Leu/Ile site in the CD loop on the gating dynamics of Kir channels and corresponding micro-structural mechanism remains unclear. Molecular dynamics simulations were performed to investigate the structural mechanism of chicken Kir2.2. Compared to WT, the I223L mutant channel bound to PIP_2_ more strongly, was activated more rapidly, and maintained the activation state more stably after PIP_2_ dissociation. Cellular electrophysiology assays of mouse Kir2.1 and human Kir2.2 indicated that, consistent with simulations, the Leu residue increased the channel responses to PIP_2_ through increased binding affinity and faster activation kinetics, and the deactivation kinetics decreased upon PIP_2_ inhibition. The Ile residue induced the opposite responses. This difference was attributed to the distinct hydrophobic side chain symmetries of Leu and Ile; switching between these residues caused the interaction network to redistribute and offered effective conformation transduction in the Leu systems, which had more rigid and independent subunits.

**Electronic supplementary material:**

The online version of this article (doi:10.1007/s12035-015-9466-x) contains supplementary material, which is available to authorized users.

## Introduction

The inward-rectifier potassium (Kir) channels conduct K^+^ ions more efficiently into the cell than in the reverse direction; these channels are involved in many physiological processes, including neuronal signaling, kidney function, insulin secretion, and heart rate control [1]. Seven subfamilies (Kir1.*x* to Kir7.*x*) with four functional groups are identified [2, 3]. The functional unit of Kir is a homomeric or heteromeric tetramer, and each monomer has a transmembrane M1-P-M2 motif (termed TMD) and a large “cytoplasmic pore” structure (termed CTD). The former contains an outer transmembrane helix (M1), an ion-selective P loop (selectivity filter, SF), and an inner transmembrane helix (M2), and the latter contains the short N-terminus and long C-terminus on the intracellular side of the membrane [2]. Specific Kir channels are regulated by different cellular factors, such as G-proteins, ATP, and pH [2]. However, phosphatidylinositol 4,5-bisphosphate (PIP_2_) is an essential factor for maintaining the activity of all Kir channels [2, 4, 5].

The function of Kir channels depends on their gating features, which is accompanied by conformational transitions. Two transmembrane locations have been proposed for the intrinsic gates: the bundle crossing with conserved hydrophobic residues formed by the M2 helix at the intracellular side and the SF at the extracellular side [3]. Another cytoplasmic gate formed by the G loop is proposed based on the crystal structures of the cytoplasmic N- and C-domains of Kir2.1 and Kir3.1 [6], Kir2.1-R218Q/T309K [7], and the Kir3.1-prokaryotic Kir channel chimera [8]. Generally, the gates formed by the transmembrane M2 and cytoplasmic G loop are considered physical gates that regulate the slow gating, and SF is considered the non-physical gate for regulating fast gating [2]. Several gating models are proposed based on the X-ray crystal structures. The combination of the X-ray crystallography and electron microscopy of KirBac3.1 suggests that bending of the M2 helix results in gate opening [9]. Reorientation and rotation of intracellular domains of KirBac3.1 are directly correlated to the ion configuration in the selectivity filter [10]. The structures of the prokaryotic KirBac1.1 and 3.1 suggest that M2 helix bending and twisting of intracellular domains are typical features of gate opening [11, 12]; this possibility is supported by the structure of Kir1.1 and corresponding experiments showing that the TM1-TM2 H bonding controls the PIP_2_ activation kinetics of Kir channels [13, 14]. The crystal structures of eukaryotic Kir3.2 and Kir2.2, as well as those of the Kir3.1-prokaryotic chimera, suggest that PIP_2_ binding would tightly couple the TMD and CTD gates for further gating [8, 15, 16]. The crystal structure of the Kir3.2 suggests that the rotation of the CTD facilitates the channel gating [17]. Although these structural and experimental results offer ample information for understanding the structure-function relationship of Kir channels, the gating kinetics, an inherently dynamic process with synergy among different gates, has not been characterized using an intuitive presentation by static structures or functional experiments.

The structure of the intracellular G loop gate is more complex than that of the transmembrane M2 gate, suggesting a more complex gating mechanism for the G loop gate and the related Kir function. A body of experimental evidence demonstrates that mutations located in the C linker [5, 16, 18, 19], the G loop itself [6], the N-terminus [5, 13, 16, 18, 19], and the CD loop [4, 5, 18, 20] significantly affect Kir functions. The corresponding structural mechanisms can be classified into two types. One is that the site regulates Kir function by binding PIP_2_ directly, and the mutation of this type of site would change both the Kir-PIP_2_ interaction affinity and Kir activation dynamics. The other is that the site does not bind PIP_2_ directly, but the mutation of this type of site would change the Kir conformation and interaction network, and further conformational transferring during gating dynamics.

For example, the CD loop is a representative loop that regulates Kir gating dynamics through the indirect transfer of conformation changes because it is not the binding site of PIP_2_ [15, 16]. The positively charged K219 in the CD loop is proposed as one binding site of the nonspecific secondary anionic phospholipid, an important composite of native cell membranes; this binding increases the PIP_2_ sensitivity of Kir2.1 channel gating [21]. The mutation of human Kir2.1 V223L in the CD loop increases both the on- and off-gating kinetics by affecting the GH loop flexibility [22]. Moreover, a conserved hydrophobic Leu/Ile residue difference in the CD loop is found in all identified human Kir channels, except Kir3.1 and Kir5.1, as indicated by the presence of Leu in the Kir1.1, Kir2.1, Kir2.4, Kir4.1, Kir4.2, and Kir7.1 channels and the presence of Ile in the Kir2.2, Kir2.3, Kir3.2, Kir3.3, Kir3.4, Kir6.1, and Kir6.2 channels [4]. Interchange mutations of this Leu/Ile site change the gating kinetics of several Kir channels in a reciprocal manner. For example, the mutation of I229 to Leu in human Kir3.4 hinders the anti-PIP_2_ antibody-induced inhibition dynamics, whereas the reverse mutation of L222 to Ile in human Kir2.1 accelerates the inhibition [20]. The mutation of I213L in human Kir2.3 exhibits a similar retarded inhibition time course as that for Kir3.4 [4]. These previous experiments suggest the key role of the conserved Leu/Ile site in the CD loop in regulating the gating dynamics of Kir channels. The CD loop regulates Kir gating through indirect pathways involving conformational changes because the CD loop is not a direct PIP_2_ binding site but forms a complex interaction network with the G loop, C linker, and N-terminus [15, 16, 18, 23, 24]. However, the intrinsic structural mechanisms of this conserved Leu/Ile site in regulating Kir gating dynamics remain unclear. Furthermore, current experimental measurements only focus on the effect of this site on the inhibition dynamics in human Kir channels; both the generality among all eukaryotic Kir channels and the activation dynamics remain unknown.

In this study, we aimed to explore the regulating role of the CD loop on Kir gating dynamics with a focus on the effect of the conserved Leu/Ile site. The structural mechanism was investigated using molecular dynamics simulations (MDS), and the gating kinetics was systematically quantified using functional measurements. The results offer insights into the regulatory effect of the conserved Leu/Ile site and the CD loop on Kir gating kinetics and further the understanding of universal Kir gating dynamics.

## Materials and Methods

### Molecular Dynamics Simulations

#### System Construction

X-ray crystal structures of the WT or I223L mutant of the chicken Kir2.2 channel in the absence or presence of PIP_2_ ligand (PDB codes: 3JYC, 3SPJ, 3SPI, and 3SPH) were employed to construct simulation systems [15, 25]. Two sets of simulation systems were established to investigate the micro-structural basis of the difference in the gating dynamics of Kir channels caused by the Leu/Ile site in the CD loop. *Set I*, the systems without the PIP_2_ ligand, contained two X-ray crystal structures of the WT (WT-3JYC) and I223L mutant (I223L-3SPJ) that originally lacked the PIP_2_ and two artificially PIP_2_-deleted structures of the WT (WT-3SPI^−^) and I223L mutant (I223L-3SPH^−^) from X-ray crystal structures of PIP_2_-Kir2.2 complexes. *Set II*, the systems in the presence of PIP_2_, contained two artificially PIP_2_-added structures of the WT (WT-3JYC^+^) and I223L mutant (I223L-3SPJ^+^) and two structures of the WT (WT-3SPI) or I223L mutant (I223L-3SPH) that were originally co-crystallized with PIP_2_. The PIP_2_ in the artificially PIP_2_-added WT (WT-3JYC^+^) and I223L mutant (I223L-3SPJ^+^) systems were separated from the corresponding PIP_2_ co-crystallized complex and were located in the target Kir channels after the superposition of the Kir2.2 structure based on the alignment of the backbones of the two transmembrane helixes. The N-terminal segment coordinates (N43-M70) of the four structures of WT-3JYC, I223L-3SPJ, WT-3JYC^+^, and I223L-3SPJ^+^ were adjusted from intrasubunit to intersubunit interaction structures by replacing the individual N-terminal segments with that of the PIP_2_ co-crystallized structures upon the alignment of the backbones of the two transmembrane helixes. This adjustment was based on the presence of N-terminal intersubunit interactions in all full-length X-ray crystal structures of both the prokaryotic and eukaryotic Kir channels, except the PIP_2_-absent chicken Kir2.2 channels [8–11, 15–17, 25]. Furthermore, the transition from the intrasubunit to intersubunit interaction state is a necessary step for Kir gating, and simulation of the spontaneous transition is beyond the capability of free equilibration MDS with limited computation power.

#### Equilibration Simulations

Each system was constructed by embedding the target Kir channel tetramer into a rectangular POPE lipid membrane plate with ~20- and 80-Å water layers capping the extracellular and intracellular sides of the membrane, respectively, and then neutralizing the system with ~100 mM sodium ions and chlorine ions to mimic the physiological ionic environment. The NAMD program [26] with the CHARMM27 all-atom force field [27] for the Kir channel and home-built parameters for the PIP_2_ ligand [23] were used for simulations with an integration time step of 1 femtosecond (fs) and periodic boundary conditions. A smooth (10–12 Å) cutoff and the particle mesh Ewald (PME) method were employed to calculate the van der Waals forces and full electrostatic interactions, respectively. Every simulation was started from energy minimization, and the constraints were gradually relieved; the simulations included 50,000 steps during which all components were fixed, except capping waters and neutralized ions, and followed by 50,000 steps with constraints on the Kir channel backbone (and heavy atoms of PIP_2_ for the PIP_2_-liganded complex systems) and the *Z*-axis of lipid phosphorus atoms, another 50,000 steps with constraints similar to the above stage but with reduced restraints on the Kir channel (only on the C_α_ atoms), and a final 50,000 steps performed with constraints only on the *Z*-axis of lipid phosphorus atoms. System heating was then performed from 0 to 310 K at 31 K increments every 10 picoseconds (ps). Finally, an unrestrained equilibration simulation of 50 nanoseconds (ns) was performed after sequential 1-ns relaxation simulations: first, both the protein backbone (and the heavy atoms of PIP_2_) and *Z*-axis of lipid phosphorus atoms were constrained, and for the second 1-ns relaxation, only the *Z*-axis of the lipid phosphorus atoms was constrained. Continuous *Z*-axis constraints were maintained on the lipid phosphorus atoms from the energy minimization and system heating to the initial 2-ns equilibration to prevent collapse of the lipid membrane before the membrane matched well to the target molecules. The 310 K heat bath was manipulated using a Langevin thermostat, and the 1 atm pressure was controlled using the Nosé-Hoover-Langevin piston method during equilibration simulations.

#### Structural Analyses

The analyses focused on two factors. First, the conformational differences or dynamics were evaluated in terms of the geometry parameters. The conformational differences in the X-ray crystal structures of the WT and I223L mutant or between the PIP_2_-absent and PIP_2_-present structures were represented as the displacement of each residue, which was defined as the distance between the heavy atom centers of each residue of the two structures when the backbones of the two transmembrane helixes were aligned. The flexibilities of both the N linker, which connected the intracellular N-terminus and the first transmembrane helix M1, and the C linker, which connected the second transmembrane helix M2 and the intracellular C-terminus, were quantified by the distance between residue R78 of the N linker and R186 of the C linker; this distance was defined as the distance between the geometrical center of the NH1 and NH2 atoms of R78 and that of R186 from the same subunit (Online Resource *ESM*_1*A*). The location of the intracellular C-terminus relative to the inner lipid membrane was defined as the distance between the D76-C_α_ atom of one subunit and the K220-C_α_ atom of the next subunit in the anticlockwise direction (Online Resource *ESM*_1*B*). The orientation of the WT I223 or mutated L223 was characterized by the angle of the I223 or L223 side chain with respect to the *Z*-axis; this angle was calculated between the vector that pointed from atom CG1 to atom CG2 of I223 (Online Resource *ESM*_1*C*) or from atom CD1 to atom CD2 of L223 (Online Resource *ESM*_1*D*) and the *Z*-axis. Second, the interaction strength between PIP_2_ and the entire Kir2.2 channel or between PIP_2_ and individual binding sites was evaluated by the non-covalent bond interactions, which included both van der Waals and electrostatic interactions. Interactions between key intra- or intersubunit residues were determined using the number of hydrogen bonds (H bonds) with the criteria that the donor-acceptor distance <3.5 Å and the donor-hydrogen-acceptor angle <45°. The system construction, structural analyses, and structure visualization were performed using the VMD program [28].

### Molecular Reconstruction

All cDNA constructs were subcloned into the pGEMHE plasmid vector and used as previously described [20]. Point mutants were produced by Pfu mutagenesis with a QuikChange kit (Stratagene, La Jolla, CA). Sequences were confirmed by DNA sequencing. Recombinant Kir2.1 and Kir2.2 WT/mutant channels and Ci-VSP were expressed in *Xenopus laevis* oocytes as described previously [20, 29]. The corresponding cRNAs were produced by T7 RNA polymerase using a kit (Promega) and were injected in quantities of 0.5–10 ng/oocyte depending on the functional expression level of the given construct.

### Electrophysiology Assay

Recordings in *X. laevis* oocytes were performed within 1 to 2 days after cRNA injection.

Whole-oocyte currents were measured using a conventional two-electrode voltage clamp (TEVC) by a Gene Clamp 500 amplifier (Molecular Devices, CA). The electrodes with a resistance less than 1 MΩ were filled with 3 M KCl dissolved in 1 % agarose to prevent the leakage of KCl into the oocytes. The cells were continuously perfused with a high-potassium solution (ND96K) containing 96 mM KCl, 1 mM NaCl, 1.8 mM CaCl_2_, 1 mM MgCl_2_, and 5 mM HEPES (pH 7.4 with KOH). The voltage protocol of 1-s sweeps composed of a 170-ms ramp from −80 to +80 mV followed by an 830-ms step to +80 mV was used to activate Ci-VSP. Additionally, the oocytes were held at −80 mV for the deactivation of Ci-VSP. Sweeps were applied until the resulting currents reached a steady state [22]. A low-potassium solution (ND96) containing 96 mM NaCl, 1 mM KCl, 1.8 mM CaCl_2_, 1 mM MgCl_2_, and 5 mM HEPES (pH 7.4 with NaOH) was used to inhibit most of the Kir currents at −80 mV. Current amplitudes were measured at −80 and +80 mV. Data acquisition was performed using pClamp 9.2 (Molecular Devices, CA).

Macropatch channel activity was recorded from devitalized oocytes under the inside-out mode of a standard patch clamp using an Axon Axopatch 200B patch-clamp amplifier and Clampex 10.0 data acquisition software (Molecular Devices). Electrodes were made from borosilicate glass using a Sutter P-97 microelectrode puller (Sutter Instrument Co., CA) that provided a tip diameter of 5–15 μm and had a resistance of 0.5–1 MΩ when filled with an electrode solution containing 120 mM KCl, 2 mM MgCl_2_, and 10 mM HEPES (pH 7.4 with KOH). Two bath solutions were used: an FVP solution containing 60 mM KCl, 5 mM EDTA-K, 5 mM KF, 0.1 mM Na_3_VO_4_, 10 mM K_4_P_2_O_7_, and 10 mM HEPES (pH 7.4 with KOH) and another FVP solution with diC_8_ PIP_2_ at 0–100 μM for dose-response measurements and at 30 μM for gating kinetics measurements. diC8 PIP_2_ was purchased from Avanti Lipids and prepared as described previously [22]. All other chemicals were purchased from Sigma. Five to seven oocytes of each batch were used for TEVC recording, three to five oocytes of the same batch were used for macropatch recordings, and three to four batches of oocytes were tested for data acquisition in each condition.

## Results

### Differences in Static Conformation Induced by the I223L Mutation in Chicken Kir2.2

Sequence alignment showed that the Leu/Ile site in the CD loop is conserved in both human (*A*) and mouse (*B*) Kir channels, and the CD loop sequence of each human Kir channel member is nearly the same as that of mouse Kir channels (Online Resource *ESM*_2), suggesting that this site would affect the Kir gating similarly for both human and mouse. Furthermore, the CD loop sequence of chicken Kir2.2 is identical to that of the human or mouse Kir2.2 channel (Online Resource *ESM*_2*B*, *last row*); therefore, the X-ray crystal structures of the PIP_2_-absent or PIP_2_-present chicken WT and I223L mutant Kir2.2 channels are ideal systems for investigating the universal structural mechanisms of the Leu/Ile site in the CD loop that affect the Kir gating in various eukaryotic species.

**Fig. 1:**
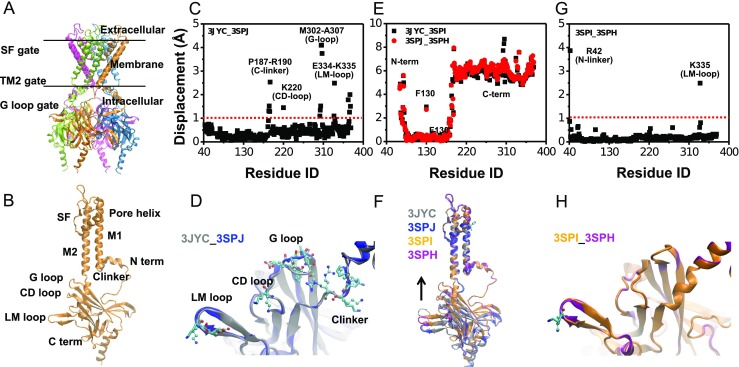
Structural features of Kir channels and differences in the static conformations of the WT and I223L mutant of chicken Kir2.2. **a**, **b** Overall functional tetramer of Kir channels with each subunit in a different color (**a**) and structural components of each subunit (**b**). **c**, **d** Conformational difference between the crystal structures of the WT (PDB code: 3JYC) and I223L mutant (PDB code: 3SPJ) in the absence of the PIP_2_; this difference was quantified using residue displacement (**c**) and is structurally illustrated in *CPK* and *Licorice* to highlight the key residues with displacements of more than 1 Å, and the overall channel is shown in *newcartoon* (**d**). **e**, **f** Conformational adjustments induced by binding of the PIP_2_ to the WT (*black*) and I223L mutant (*red*) (**e**) and the corresponding structural diagrams (**f**). **g**, **h** Conformational difference between the crystal structures of the WT (PDB code: 3SPI) and I223L mutant (PDB code: 3SPH) in the presence of PIP_2_ shown as a quantification of the residue displacement (**g**) and an intuitive presentation of the conformational superposition (**h**). The residue displacement was defined as the distance between the heavy atom centers of each residue between the two structures after alignment of the two transmembrane helixes

The intrinsic structural difference induced by the conserved Leu/Ile site is a prerequisite for triggering the gating kinetics differences of Kir channels. The individual residue displacements of chicken Kir2.2 structures indicated that the conformational differences between the PIP_2_-absent WT-3JYC and I223L-3SPJ mutant structures were centered in the intracellular C-terminal domain with residue displacements equal to or greater than 1 Å (Fig. 1a, b), especially in the following regions: C linker P187-R190, CD loop K220, G loop M302-A307, and LM loop E334-K335 (Fig. 1c, d). The quantitative comparisons between the PIP_2_-absent and PIP_2_-present WT (*black*) or I223L mutant (*red*) structures (Fig. 1e) and the visualizations of the structures (Fig. 1f) clearly indicated that PIP_2_ binding induced an upward movement of the intracellular N- and C-termini toward the transmembrane direction in both structures. The PIP_2_ binding also reduced the conformational difference between the WT and I223L mutant: the displacements of individual residues decreased (Fig. 1g), and the structures were further superimposed (Fig. 1h). The conformational difference between the PIP_2_-absent WT-3JYC and I223L-3SPJ mutant channels suggested the different PIP_2_ binding ability or the ability to transduce conformational changes. The decreased conformational difference in the PIP_2_-present structures compared with the PIP_2_-absent structures indicated that the residues at these sites play a key role in Kir gating dynamics. Thus, detailed dynamic features are required to understand the structural mechanism underlying the regulation of Kir gating by the Leu/Ile site.

### PIP_2_ Binding Difference Induced by the I223L Mutation in Chicken Kir2.2

**Fig. 2:**
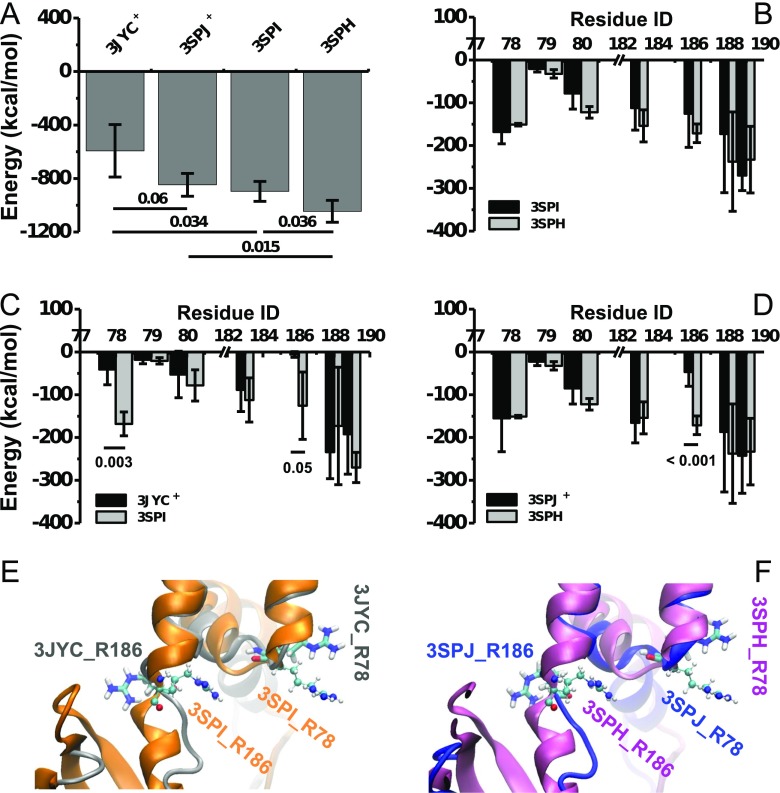
Channel-PIP_2_ interaction difference between the WT and I223L mutant of chicken Kir2.2. Global PIP_2_ binding energies of the artificially PIP_2_-added WT (WT-3JYC^+^) and I223L mutant (I223L-3SPJ^+^) systems and of the PIP_2_ co-crystallized WT (WT-3SPI) and I223L mutant (I223L-3SPH) systems are presented in **a**. Comparisons of the energy distributions among each PIP_2_-binding site between the WT-3SPI and I223L-3SPH systems, the WT-3JYC^+^ and WT-3SPI systems, and the I223L-3SPJ^+^ and I223L-3SPH systems are shown in **b**, **c**, and **d**, respectively. Orientation differences of the residues R78 and R186 between the PIP_2_-absent (*Licorice*) and PIP_2_-present (*CPK*) crystal structures of the WT-3JYC and I223L-3SPJ mutant are illustrated in **e** and **f**, respectively. The interaction energy includes non-covalent van der Waals and electrostatic interactions, and the global energy or distribution of each binding site for each system is presented as the mean ± SD of the tetramer during the last 10 ns of the equilibration simulation. Only a partial subunit of the Kir channel is shown in *newcartoon* with different *colors* for clarity

MD equilibration simulations were performed using the systems as labeled in Table 1 to investigate the dynamic characteristics of chicken Kir2.2. Because the PIP_2_ binding strength is a key factor in Kir gating function, the Kir2.2-PIP_2_ interactions were first evaluated for the four PIP_2_-liganded complex systems. The results indicated a weaker interaction energy in the WT channel than in the I223L mutant in both the artificially PIP_2_-added systems (*left two bars*) and the PIP_2_ co-crystallized systems (*right two bars*) (Fig. 2a). Furthermore, the binding energy of the artificially PIP_2_-added complex was weaker than that of the PIP_2_ co-crystallized complex for both the WT (*the first and third bars*) and I223L mutant (*the second and fourth bars*) systems (Fig. 2a). The global H bond interactions of the four complexes were consistent with the non-covalent interactions (Online Resource *ESM*_3*A*), implying that the electrostatic interactions dominated the PIP_2_-Kir2.2 interaction.

**Table 1:** Summary of simulation setup

Set	System	Free MD duration (ns)	Description
PIP_2_ absence	WT-3JYC	50	Crystallized WT
	I223L-3SPJ	50	Crystallized I223L mutant
	WT-3SPI^−^	50	PIP_2_ deleted WT
	I223L-3SPH^−^	50	PIP_2_ deleted I223L mutant
PIP_2_ presence	WT-3JYC^+^	50	PIP_2_ added WT
	I223L-3SPJ^+^	50	PIP_2_ added I223L mutant
	WT-3SPI	50	Crystallized WT with PIP_2_ ligand
	I223L-3SPH	50	Crystallized I223L mutant with PIP_2_ ligand

Further analysis showed that each PIP_2_ bound a Kir2.2 monomer independently with coincident binding sites of R78, W79, R80, K183, R186, K188, and K189 (Online Resource *ESM*_3*B*); these sites are all positively charged basic amino acids, except for W79, and are located near the inner membrane for effective binding to the negatively charged PIP_2_ polar head. The energy distribution among the binding sites did not significantly differ between the PIP_2_ co-crystallized WT-3SPI (*black*) and I223L-3SPH (*gray*) mutant systems (Fig. 2b), although the global PIP_2_-Kir2.2 interaction energy of the I223L mutant system was stronger than that of the WT system with *p* = 0.036. The PIP_2_ ligand bound R78 (*p* = 0.003) and R186 (*p* = 0.05) more tightly in the PIP_2_ co-crystallized WT-3SPI systems (*gray*) than those in the artificially PIP_2_-added WT-3JYC^+^ systems (*black*) (Fig. 2c), and only the binding energy for R186 significantly differed (*p* < 0.001) between the PIP_2_ co-crystallized I223L-3SPH (*gray*) and artificially PIP_2_-added I223L-3SPJ^+^ (*black*) mutant systems (Fig. 2d).

The stronger global interaction energy of the I223L mutant channel in both the PIP_2_ co-crystallized and artificially PIP_2_-added systems suggested that the I223L Kir2.2 had higher PIP_2_ sensitivity and could more effectively gate the Kir channel. The energy difference between the PIP_2_ co-crystallized and artificially PIP_2_-added Kir2.2 channels for both the WT and I223L mutant systems indicated that PIP_2_ binding requires time to induce the conformational change for optimal interaction. Furthermore, the energy distributions revealed that there were two sites, R78 and R186, for the WT channel but only one site, R186, for the I223L mutant channel that significantly differed between the artificially PIP_2_-added and co-crystallized systems. This finding suggested a weaker response of the WT channel to the PIP_2_ ligand than that of the I223L mutant, and the R78 and R186 sites played key roles in the PIP_2_-induced conformational regulation. Superposition of the PIP_2_-absent and PIP_2_-present X-ray crystal structures of both the WT (Fig. 2e) and the I223L mutant (Fig. 2f) systems showed that PIP_2_ binding induced reorientation of both the R78 and R186 side chains; the two chains initially pointed away from each other (*Licorice*) but then assume an approximately parallel orientation (*CPK*) with a reduced distance (Fig. 2e, f). Thus, the reorientation dynamics and extent could be important indicators for evaluating the PIP_2_-induced regulation of the conformation.

### Dynamic Differences in Conformation Induced by the I223L Mutation upon PIP_2_ Binding in Chicken Kir2.2

**Fig. 3:**
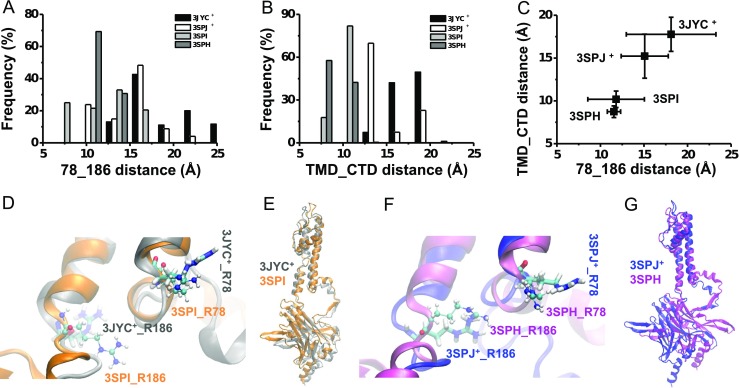
Dynamic conformation differences between the PIP_2_-present WT and I223L mutant of chicken Kir2.2. Distributions of the R78-R186 distance and TMD-CTD distance for systems of artificially PIP_2_-added WT (WT-3JYC^+^, *black*) and I223L mutant (I223L-3SPJ^+^, *white*) and of the PIP_2_-co-crystallized WT (WT-3SPI, *gray*) and I223L mutant (I223L-3SPH, *dark gray*) are shown in **a** and **b**, respectively. The correlation is shown in **c** with the means ± SD. The conformational comparisons with emphases on the orientations of R78 and R186 (**d**, **f**) and the upward motions of the CTD domains (**e**, **g**) for both the WT (**d**, **e**) and I223L mutant (**f**, **g**) systems are shown in (**d**–**g**). The R78-R186 distance was defined as the distance between the geometrical center of the NH1 and NH2 atoms of R78 and that of R186 from the same subunit. The TMD-CTD distance was defined as the distance between the D76-C_α_ and K220-C_α_ from the adjacent subunit in the clockwise direction. Only the data from the last 10 ns of equilibration of each system are shown here, and the average in **c** was obtained from the tetramer after the last 10-ns trajectory average of each subunit. Subunit A in the 50-ns snapshot of each system was used for conformational comparisons in **d**–**g** with a presentation similar to that in Fig. 2

Comparisons of the static conformations of chicken Kir2.2 showed the upward movement of the intracellular CTD and reorientation of the R78 and R186 side chains upon PIP_2_ binding. The PIP_2_-Kir2.2 interaction energy analysis also provided the cue that the reorientations of R78 and R186 could be the trigger point for further regulation of the conformation. Here, we further investigated the dynamics of the reorientations of both R78 and R186 and of the upward movements of the intracellular CTD of all four PIP_2_-bound systems during 50-ns equilibration processes. The evolution of the former, as characterized by the R78-R186 distance, showed that the artificially PIP_2_-added WT-3JYC^+^ (Online Resource *ESM*_4*A*) and I223L-3SPJ^+^ mutant (Online Resource *ESM*_4*B*) systems had larger fluctuations than those of the PIP_2_ co-crystallized systems (Online Resource *ESM*_4*C*-*D*); in contrast, the I223L mutant (Online Resource *ESM*_4*B*, *D*) had a smaller fluctuation than that of the WT channel (Online Resource *ESM*_4*A*, *C*) for both systems. In particular, the artificially PIP_2_-added I223L-3SPJ^+^ mutant system showed a relatively consistent trend of the four subunits and smaller R78-R186 distances (Online Resource *ESM*_4*B*) than the WT-3JYC^+^ system (Online Resource *ESM*_4*A*). The statistics of the last 10-ns equilibration processes revealed the distributions of the R78-R186 distance (Fig. 3a), which showed that the I223L mutant system responded more easily to the PIP_2_ ligand, leading to a smaller R78-R186 distance. The greater stability of the distribution of the I223L-3SPH system also implied that the I223L mutant system would remain in the stable state after PIP_2_ induction. The superposition of the 50-ns snapshots provided an intuitive illustration of the extent of the orientation regulation of R78 and R186 upon PIP_2_ binding in the WT (Fig. 3d) and I223L mutant (Fig. 3f) systems.

The evolution of the TMD-CTD distance exhibited a trend similar to that of the R78-R186 distance. After 50 ns of equilibration, only one subunit of the artificially PIP_2_-added WT-3JYC^+^ system showed an upward movement with a reduced TMD-CTD distance, and for the other three subunits, the TMD-CTD distances fluctuated around the initial value (Online Resource *ESM*_5*A*). In contrast, the I223L-3SPJ^+^ system showed contrasting behavior with a reduced TMD-CTD distance for three subunits (Online Resource *ESM*_5*B*), as expected. Comparatively, the WT-3SPI (Online Resource *ESM*_5*C*) and I223L-3SPH (Online Resource *ESM*_5*D*) systems had a more stable TMD-CTD distance, with the latter system having a slightly smaller TMD-CTD distance. The statistics for the last 10 ns of equilibration showed the difference in the TMD-CTD distance distributions (Fig. 3b), implying that the upward movement of the intracellular CTD upon PIP_2_ binding occurred more rapidly in the I223L mutant system with a smaller TMD-CTD distance than in the WT system. The difference between WT-3SPI and I223L-3SPH indicated that the PIP_2_-bound state was more stable in the I223L mutant system than in the WT system. The superposition of the 50-ns snapshots is presented in an intuitive manner in Fig. 3e, g. The relevance of the reorientation of R78 and R186 residues to the upward movement of the intracellular CTD upon PIP_2_ binding is shown in Fig. 3c. Taken together, these results showed that a smaller R78-R186 distance corresponded to a smaller TMD-CTD distance, which indicated the triggering role of the side chain reorientation of R78 and R186.

### Difference in the Interaction Network Induced by the I223L Mutation in Chicken Kir2.2

**Fig. 4:**
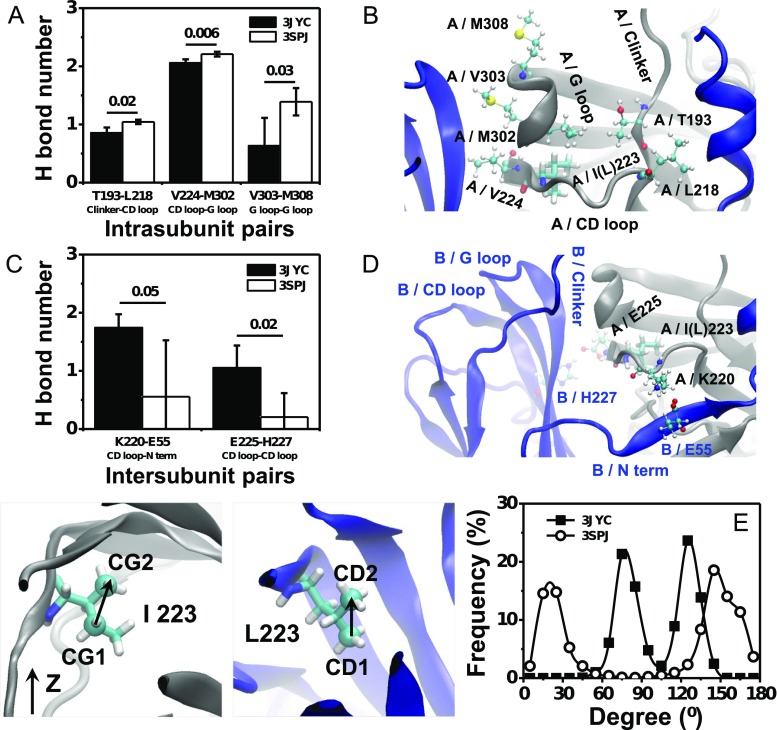
Difference in the interaction networks of the PIP_2_-absent WT-3JYC and I223L-3SPJ mutant of chicken Kir2.2. Key amino acid pairs of the intrasubunit interaction with significantly different H bond interactions (**a**) and the corresponding location (**b**) are shown. Those of the intersubunit interaction are presented in **c**, **d**. The distributions of the sidechain orientation of I223 and L223 are shown in **e** with a bin size of 15°. The H bond interactions are presented as the mean ± SD of the tetramer after the last 10-ns trajectory average of each subunit. The key amino acids are presented as *CPK* with labeling of the subunit and the corresponding secondary structures. I(L)223 is shown in *Licorice* for clarity

The differences between the static conformation of the WT and I223L mutant channels, the PIP_2_ binding energy, and the conformational dynamics of PIP_2_-liganded systems supported our hypothesis that the I223L mutation-triggered change in the conformation of the Kir channel would also change the PIP_2_ binding affinity and conformational dynamics. However, the conformational dynamics upon PIP_2_ binding are determined by both the PIP_2_ binding strength and interaction network of the Kir channel; the former determines the driving force, and the latter governs the ability to transduce conformational changes. Although the aforementioned results demonstrated that the I223L mutant resulted in stronger PIP_2_ binding energy and more rapid conformational dynamics, the issues of whether the I223L mutation changes the interaction network of the Kir channel itself and the contributions of the interaction network to the conformational dynamics remain unclear. To answer these questions, we investigated the intra- and intersubunit interaction networks to evaluate the intrinsic conformational difference between the PIP_2_-absent WT-3JYC and I223L-3SPJ mutant channels. The results showed that the I223L mutant channel had stronger intrasubunit interactions than those of the WT with a significantly higher number of H bonds; these H bonds were involved in the interactions between T193 of the C linker and L219 of the CD loop, V224 of the CD loop and M302 of the G loop, and V303 of the G loop and M308 of the G loop (Fig. 4a, b). In contrast, the I223L mutant channel had weaker intersubunit interactions than those of the WT, which mainly resulted from the amino acid pairing between K220 of the CD loop and E55 of the N-terminus and between E225 of the CD loop and H227 of the CD loop (Fig. 4c, d). These results suggested that due to the stronger intrasubunit interactions and weaker intersubunit interactions, each subunit of the I223L mutant channel moved more independently for a quick response to PIP_2_ binding. These features were reflected intuitively by the higher fluctuation of the residue displacement of WT-3JYC subunits (Online Resource *ESM*_6*A*) and the smaller residue displacement of the I223L-3SPJ mutant subunits (Online Resource *ESM*_6*B*). The three strengthened specific amino acid pairs intimately linked the PIP_2_ binding site, CD loop, and G loop; this result further supported our hypothesis that the binding of PIP_2_ near the inner membrane would transfer to the gating of the intracellular G loop through the interaction network. The more favorable network for transducing conformational changes and the stronger PIP_2_ binding energy suggested that Kir channels with a Leu residue in the CD loop would have more rapid opening dynamics than those of the channels with the Ile residue.

Ile and Leu are both hydrophobic residues with a similar side chain size but different symmetries. How can such minor changes cause distinct conformational differences? One possibility is that the difference in the side chain symmetry caused a difference in the surrounding environment, which subsequently resulted in a distinct interaction network. The quantification of the orientation of the Ile or Leu side chain relative to the *Z*-axis confirmed our conjecture. The results demonstrated that the orientation of the Ile side chain of the WT channel was nearly perpendicular to the *Z*-axis with main distributions around 75° and 125° (Fig. 4e, squares), whereas the Leu side chain of the I223L mutant channel was nearly parallel to the *Z*-axis with main distributions around 20° and 145° (Fig. 4e, cycles).

The above simulation analyses indicated that the mutation of the Leu/Ile site in the CD loop would result in a different surrounding environment with a distinct side chain symmetry; thus, the mutation would lead to different intra- and intersubunit interaction networks for the Kir channel and further induce the transduction of distinct conformational changes during the activation process when PIP_2_ binds.

### Differences in Conformational Recovery Induced by the I223L Mutation in Chicken Kir2.2

**Fig. 5:**
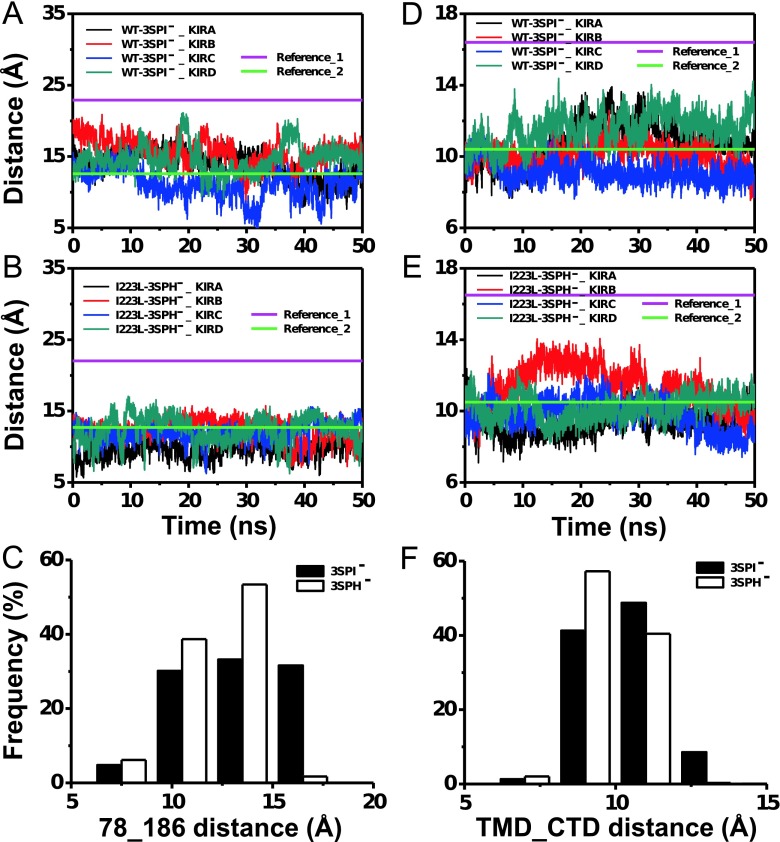
Evolution of the R78-R186 distance and TMD-CTD distance in PIP_2_-deleted WT-3SPI^−^ and I223L-3SPH^−^ mutant systems of chicken Kir2.2. The evolutions of the R78-R186 distance (**a**, **b**) and TMD-CTD distance (**d**, **e**) of the WT (**a**, **d**) and I223L mutant (**b**, **e**) are presented for each subunit. The corresponding distributions are shown in **c** and **f**, respectively. The data of **c**, **f** were collected from the tetramer during the last 10 ns of equilibration. References 1 and 2 for the R78-R186 distance (**a**, **b**) or TMD-CTD distance (**d**, **e**) are the corresponding distances in the crystal structures of the PIP_2_-absent (*Reference_1*) and PIP_2_-present (*Reference_2*) WT and I223L mutant

Deactivation dynamics after PIP_2_ dissociation is another important parameter that describes Kir channel function. Similar to the activation dynamics, the evolution of both the R78-R186 distance and TMD-CTD distance during the 50-ns equilibration processes after PIP_2_ dissociation was calculated to evaluate the recovery dynamics. The results demonstrated that the fluctuations of both the R78-R186 distance (Fig. 5a) and TMD-CTD distance (Fig. 5d) in the WT-3SPI^−^ system were larger than those of the I223L-3SPH^−^ mutant system (Fig. 5b, e). The statistics of the last 10-ns equilibration revealed that the distribution of the R78-R186 distance of the I223L-3SPH^−^ mutant system (*white*) was clustered in the lower range with a higher frequency than that of WT-3SPI^−^ (*black*) (Fig. 5c), and the distribution of the TMD-CTD distance followed a similar trend (Fig. 5f). These results implied that the I223L mutant system likely kept the channel in the activated state longer than the WT system did even after the dissociation of the PIP_2_. Furthermore, the fluctuation of the R78-R186 distance (Fig. 5a, b) was larger than that of the TMD-CTD distance (Fig. 5d, e), which indicated that the reorientation of R78 and R186 triggered the activation or deactivation of Kir channels upon PIP_2_ association or dissociation, respectively.

Considering all of the structural analyses, we hypothesized that the channels with Leu in the CD loop had higher PIP_2_ sensitivity with stronger PIP_2_ binding energy, faster activation dynamics induced by the increased capability to transduce conformational changes with greater independence in the subunit movement, and slower deactivation dynamics with a stable intrasubunit interaction network.

### Gating Kinetics Difference Induced by the Leu/Ile Site Mutation in Both Mouse Kir2.1 and Human Kir 2.2

**Fig. 6:**
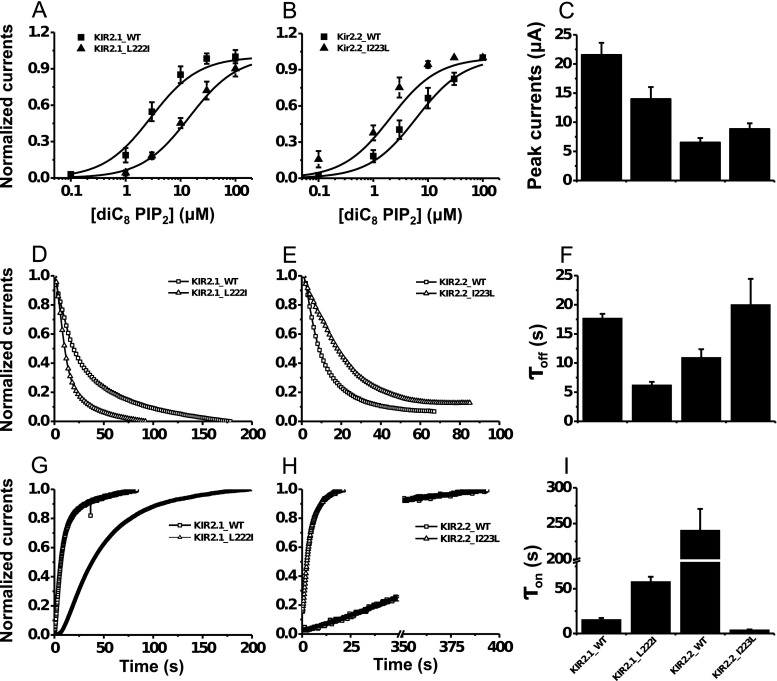
Differences in the gating kinetics of mouse Kir2.1 and human Kir2.2 induced by the mutual mutation of the Ile/Leu site in the CD loop. Dose-response curves (**a**, **b**), PIP_2_ antibody inhibition trace (**d**, **e**), and PIP_2_-induced gating trace (**g**, **h**) of the mouse Kir2.1 WT and L222I mutant (**a**, **d**, **g**) and the human Kir2.2 WT and I223L mutant (**b**, **e**, **h**) were experimentally measured. The corresponding summary data of the peak current, the inhibition time constant *τ*_off_, and the recovery time constant *τ*_on_ are provided in **c**, **f**, and **i**, respectively. *τ*_off_ represents the time needed for the PIP_2_ antibody to inhibit the Kir channel current to half of its initial amplitude, and *τ*_on_ represents the time needed for PIP_2_ to increase the Kir current to half of its maximum amplitude. *Solid lines* are the Hill fitted lines to the data points of the Kir channels. Each *data point* is the average of five to six cells from at least three independent experiments. The dose-response curve and all summary data are expressed as the mean ± SE, and the inhibition and activation trace data are shown with only the mean values for clarity

To verify the micro-structural predictions of the regulatory role of the Leu/Ile site on Kir gating dynamics, we systematically measured the electrophysiological difference in the gating dynamics between the WT and Leu/Ile mutant for both mouse Kir2.1 and human Kir2.2 channels (Online Resource *ESM*_2). The diC8 PIP_2_ dose-response curve was right shifted for the L222I mutant of Kir2.1 with an increase in the EC_50_ of 14.01 ± 0.98 μM relative to the 2.89 ± 0.31 μM for the WT channel (Fig. 6a). In contrast, the dose-response curve of the Kir2.2 I223L mutant was left shifted with a decreased EC_50_ of 1.90 ± 0.23 μM relative to the 6.14 ± 0.45 μM of the WT channel (Fig. 6b). Moreover, the whole-cell peak current of the Kir2.1 L222I mutant decreased to 14.00 ± 2.03 μA from the 21.57 ± 2.05 μA of the WT channel and that of the Kir2.2 I223L mutant increased to 8.86 ± 0.95 μA from the 6.56 ± 0.73 μA of the WT channel (Fig. 6c). These results demonstrated a stronger interaction between Kir channels and PIP_2_ when a key Leu residue was present. The discrepancy of EC_50_ and whole-cell current between Kir2.1 and Kir2.2 maybe resulted from different species or not controlled expression during plasmid transfection.

Voltage-activated lipid phosphatase Ci-VSP should reduce the PIP_2_ level in intact cells and thus inhibit Kir currents [29]. The inhibition rate depends on both the strength of the channel-PIP_2_ interaction and the recovery ability of the channel itself. Compared with the WT Kir2.1 channel, the L222I mutant channel demonstrated accelerated closing dynamics (Fig. 6d) in response to activated Ci-VSP, with a shortened *τ*_off_ of 6.26 ± 0.51 s from the 17.73 ± 0.72 s (Fig. 6f, left two bars), and a slowed re-opening dynamics (Fig. 6g) after the inactivation of Ci-VSP, with a prolonged *τ*_on_ of 58.12 ± 5.18 s from the 15.72 ± 1.66 s of the WT channel (Fig. 6i, left two bars). Further supporting the key role of Leu/Ile in the channel-PIP_2_ interaction, the reverse mutation of Kir2.2 I223L demonstrated reversed trends of delayed closing dynamics (Fig. 6e) with increased *τ*_off_ (Fig. 6f, right two bars) and faster opening dynamics (Fig. 6h) with decreased *τ*_on_ (Fig. 6i, right two bars).

The quantitative measurements were consistent with the above conformational predictions, i.e., a higher binding energy with faster reorientations of the R78 and R186 side chains and upward movements of CTD upon PIP_2_ binding corresponded to higher PIP_2_ sensitivity and faster opening dynamics, and the more stable conformations after PIP_2_ dissociation corresponded to slower closing dynamics for the channels with the Leu site than those with the Ile site.

### The Key Role of the CD Loop as a Bridge Between the C Linker and the G Loop That Regulates Kir Gating Kinetics

**Fig. 7:**
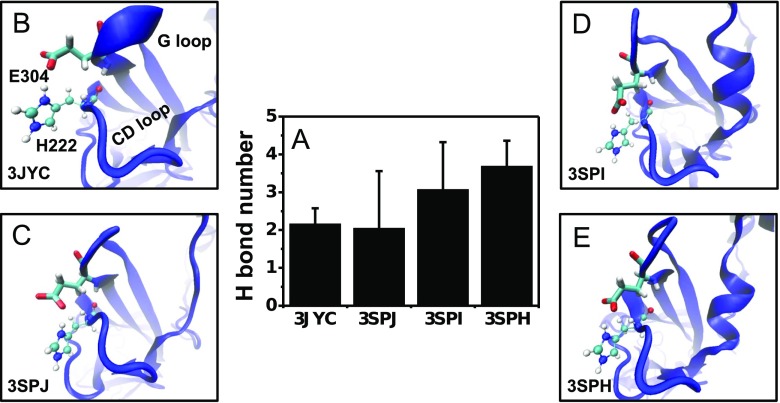
Comparison of the intrasubunit H222-E304 interaction between the PIP_2_-absent and PIP_2_-present Kir2.2 WT and I223L mutant systems. The quantified *H bond numbers* are presented in **a** as the mean ± SD of the tetramer after the last 10-ns trajectory average of each subunit, and the visual conformations of the 50-ns snapshot are presented in **b**–**e** for the PIP_2_-absent WT-3JYC (**b**) and I223L-3SPJ mutant (**c**) systems and for the PIP_2_-present WT-3SPI (**d**) and I223L-3SPH mutant (**e**) systems, respectively, with *blue* *newcartoon* for the Kir channel subunit and *Name CPK* for H222 and *Licorice* for E304

**Fig. 8:**
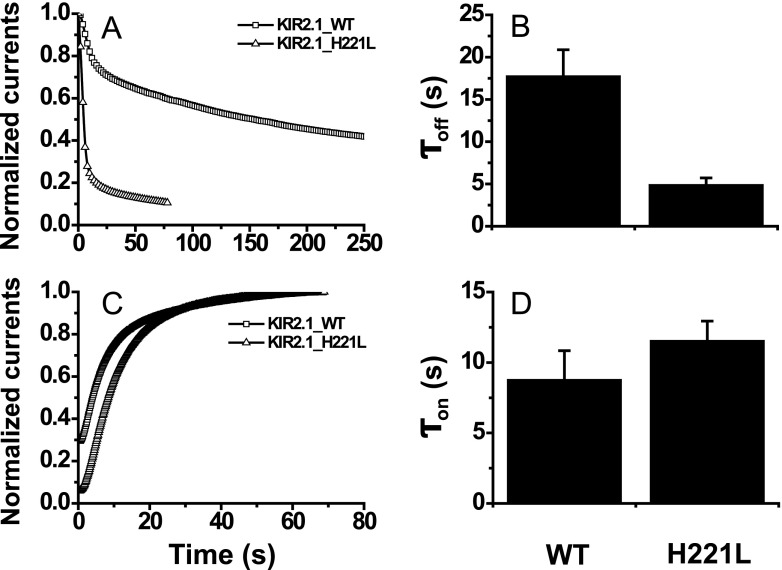
Differences in the gating kinetics of mouse Kir2.1 induced by the mutation of H222L in the CD loop. The PIP_2_ antibody inhibition trace (**a**) and PIP_2_-induced gating trace (**c**) of mouse Kir2.1 WT (*squares*) and H222L mutant (*triangles*) were experimentally measured. The corresponding summary data of the inhibition time constant *τ*_off_ (**b**) and recovery time constant *τ*_on_ (**d**) are also provided. The *solid lines* are the Hill fitted lines to the data points of the Kir channels. Each *data point* is the average of five to six cells from at least three independent experiments, and the inhibition and activation trace data are shown with only the mean values for clarity. The summary data are expressed as the mean ± SD

The mutation from Ile to Leu strengthened the intrasubunit interaction network (Fig. 4a, b), which offered an effective pathway for transferring conformation changes from the C linker to the G loop gate through the CD loop. The importance of the V224-M302 interaction between the CD loop and G loop is verified in Kir2.1 channels; the V223L mutation (this site corresponds to V224 in Kir2.2) accelerates both the inhibition and activation dynamics [22]. In fact, another residue pair, H222 and E304, provided a strong interaction between the CD loop and G loop in addition to the interaction of the V224-M302 pair. The simulations demonstrated that both the WT and I223L mutant channels had stable H222-E304 interactions with comparable numbers of H bond and that the interactions increased slightly upon PIP_2_ binding (Fig. 7a). The conformations showed that the interactions resulted from both backbone and side chain electrostatic interactions (Fig. 7b–e). According to the above prediction that the interaction between the CD loop and G loop would favor transduction of the conformational change to the G loop gate upon PIP_2_ binding, the disruption of the H222-E304 interaction would weaken the interaction between the CD loop and G loop and further affect the Kir gating kinetics. The experimental measurements of the corresponding H221L mutation in mouse Kir2.1 channels proved our hypothesis, and the results showed a trend that was similar to that for the mutation from Leu to Ile, which led to faster inhibition dynamics (Fig. 8a, b) and slower activation dynamics (Fig. 8c, d). These results further support the key role of the CD loop as a bridge between the C linker and G loop that regulates Kir gating kinetics. However, the central location of the CD loop also introduced complexity. Our work indicated that the mutation of Leu to Ile in the conserved site of the CD loop would slow down the activation dynamics and accelerate the inhibition dynamics through a weakened intrasubunit interaction between the CD loop and G loop (Fig. 6), and the mutation of the residue on the front site exhibited a similar trend (Fig. 8). The comparison between the slight increase of *τ*_on_ and the significant decrease of *τ*_off_ hinted that H221L mutation induced more extensive interaction network change but not just the interaction between the CD loop and G loop. However, the mutation of a residue on the back site would accelerate both the activation and inhibition dynamics [22]. Thus, the cooperative regulation by the key residue along with the residues on the front and back sites should be considered in the channel gating.

## Discussion

The goal of this study was to elucidate the structural basis of the gating dynamics of Kir channels. Sequence alignment suggested conservation of the Leu/Ile site in the CD loop among eukaryotic Kir channels (Online Resource *ESM*_2). Although this conservation is correlated to its role in the inhibition kinetics of the Kir channel, which was preliminarily described in the literatures, the generalization of the effect of this site on gating kinetics and the structural mechanism remain unclear. In this study, MD simulations on chicken Kir2.2 and the quantified experiments on both mouse Kir2.1 and human Kir2.2 channels were performed in order to understand the micro-structural mechanism of this conserved site and the CD loop in regulating Kir gating dynamics. The micro-structural and dynamic results exhibited by the MD simulations in this study that cannot be obtained by either experimental measurements in the molecular level or static X-ray structures in the atomic level offered new insights into understanding the gating mechanism of Kir channels.

Compared with the amino acids involved in binding PIP_2_ directly, the amino acids that do not bind PIP_2_ directly introduce more complexity to the gating mechanism. The conserved Leu/Ile site in the CD loop does not bind PIP_2_ directly; instead, this site has a distinct effect on the channel-PIP_2_ interaction and channel function, and this effect serves as one of the mechanistic bases for the distinct channel-PIP_2_ interactions of nearly all eukaryotic Kir channels. Both Leu and Ile are hydrophobic residues with the same length of side chain, and interact less with surrounding residues. The reason for the remarkable impact of this type of residue is its key location in the CD loop, which interacts intimately with the C linker, N-terminus, and G loop [15, 25, 30]. Because of this special location, the CD loop acts as the bridge that connects the PIP_2_ binding sites and intracellular G loop gate. The distinct side chain symmetry of the hydrophobic Leu/Ile residues would affect both the nearby hydrophobic environment and interaction network; these effects cause differences in the capability to transduce conformational changes and/or in the PIP_2_ binding affinity and are followed by significant changes in the gating kinetics of the Kir channel. In fact, different hydrophobic amino acids affect the function of distinct proteins, e.g., the mutation of the conserved Ala site in helix 2 of the rice plasma membrane intrinsic protein to Ile/Val increases the water permeability, whereas the reverse mutation decreases the water permeability [31], and the hydrophobic contacts mediated by different hydrophobic residues between the center of the βI domain and the α_1_/α_7_ helices regulate the conformation of integrin α_4_β_7_ [32]. Our findings provided a novel mechanism by which the conserved, distant residue site can regulate Kir gating via regulating both PIP_2_ binding ability and the capability to transduce conformational changes. The importance of the capability to transduce conformational change through a complicated interaction network for Kir gating dynamics emphasized in this study is not contradictory with the models proposed by Hansen et al. [15] or by Clarke et al. [10], because the efficiency of both the upward movement and the rotation of CTD for gate opening depends on the capability of conformational transfer.

The simulations in this study predicted the importance of R186 in the C linker through dual actions on the direct binding site of PIP_2_ and the dynamic regulation that transduces conformational changes; these findings are consistent with the experiments showing that the K185Q mutant of Kir2.1 (corresponding to the R186 site of Kir2.2) reduces the current and accelerates PIP_2_ antibody inhibition [5]. The inactivation without the upward motion of the CTD domains in a short-chain derivative of pyrophosphatidic acid (PPA)-liganded chicken Kir2.2 indicated the importance of the C linker for Kir gating because PPA cannot bind the C linker completely due to the lack of an inositol ring [15]; this result is consistent with a recent functional study showing that anionic lipids with a small head group failed to activate Kir channels in the absence of PIP_2_ [33]. The rotation or upward movement of the intracellular domains of each subunit is a key process in current Kir channel activation models [8, 15–17]. The increased rigidity and independence of each subunit induced by the Leu/Ile mutation with the redistribution of the intra- and intersubunit interaction networks provide Kir channels with favorable features for the rotation or upward movement of intracellular domains.

The PIP_2_-induced gating of the Kir channel is an intrinsically dynamic process and is difficult to understand thoroughly using static crystal structures. MDS is an ideal tool for studying the dynamics along with the conformational regulation. The PIP_2_-driven gating of Kir channels occurs on the microsecond timescale with large conformational changes, which is beyond the capability of MDS, especially for equilibration simulations. However, MDS offer valuable information for understanding the structure-function relationship of Kir channels. An intermediate state of the cytosolic G loop gate between the closed and open conformations was proposed from MD simulations [23]. Although the gating process was not simulated, the movement of the CTD toward or away from the inner membrane, the key step in Kir activation [34], was visualized in this study. The correlation among the reorientation of the R78 and R186 side chains, the global motion of the CTD, the redistributed intra- and intersubunit interaction networks, and the correspondence between electrophysiological experiments and MD simulations led to a structural mechanism for interpreting and understanding the key role of the conserved site and the CD loop in the Kir gating dynamics.

## Electronic Supplementary Material

Below is the link to the electronic supplementary material.

ESM 1 (PDF 748 kb)
