# Supplementary material for: Structural Basis for Differences in Dynamics Induced by Leu Versus Ile Residues in the CD Loop of Kir Channels
Source: Mol Neurobiol. 2015 Oct 31;53(9):5948–61. doi: 10.1007/s12035-015-9466-x (PMC5085999; doi:10.1007/s12035-015-9466-x)
Supplement: Supplementary file 1 — (PDF 748 kb) [file 12035_2015_9466_MOESM1_ESM.pdf]

Structural basis for differences in dynamics induced by Leu versus Ile residues in the CD loop of Kir channels

Shouqin Lü[1,2,3,ξ], Hailong An[4,ξ], Hailin Zhang[5,*], Mian Long[1,2,3,*]

[1]Center of Biomechanics and Bioengineering, [2]Key Laboratory of Microgravity (National Microgravity Laboratory) and [3]Beijing Key Laboratory of Engineered Construction and Mechanobiology, Institute of Mechanics, Chinese Academy of Sciences, Beijing 100190, China. [4] Key Laboratory of Molecular Biophysics, Hebei Province, Institute of Biophysics, School of Sciences, Hebei University of Technology, Tianjin 300401, China. [5]Key Laboratory of Neural and Vascular Biology, Ministry of Education, The Key Laboratory of Pharmacology and Toxicology for New Drug, Hebei Province, Department of Pharmacology, Hebei Medical University, Shijiazhuang 050017, China.

[ξ] Shouqin Lü and Hailong An contributed equally to this work.

[*]Address correspondence to:

Dr. Hailin Zhang, Tel. 86-311-86265562, Fax. 86-311-86265562, zhanghl@hebmu.edu.cn, Hebei Medical University, Shijiazhuang 050017, China, or Dr. Mian Long, Tel. 86-10-82544131, Fax. 86-10-82544131, mlong@imech.ac.cn, Institute of Mechanics, Chinese Academy of Sciences, Beijing 100190, China.

Supplementary material

ESM_1: Schematic of definitions for R78-R186 distance, TMD-CTD distance, and sidechain orientations of Ile and Leu residues.

ESM_2: Sequence alignments of the CD loop for human and mouse Kir channels.

ESM_3: Global Hbond interactions and the distributions among binding sites for PIP_2-liganded chicken Kir2.2 systems

ESM_4: Evolution of R186-R78 distance for PIP_2-liganded chicken Kir2.2 systems.

ESM_5: Evolution of TMD-CTD distance for PIP_2-liganded chicken Kir2.2 systems.

ESM_6: Conformational stability of PIP_2-absent chicken Kir2.2 WT and I223L mutant systems

ESM_1

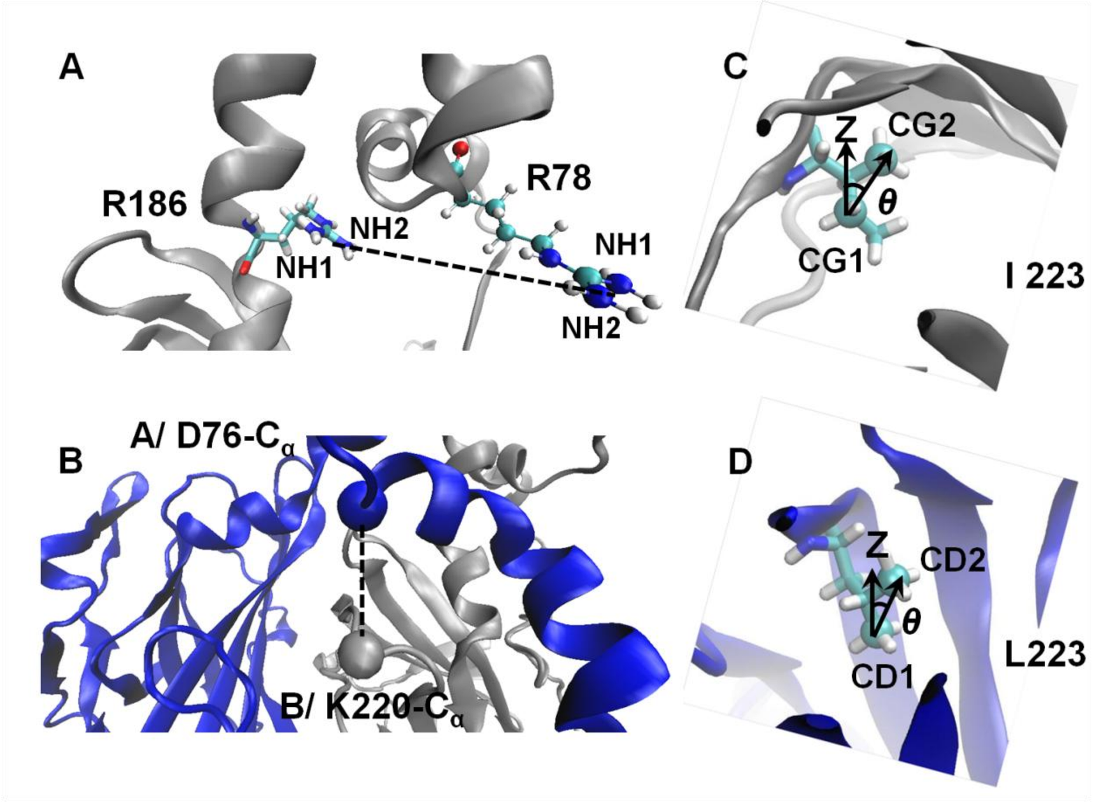

ESM_1: Schematic of definitions for R78-R186 distance (*A*), TMD-CTD distance (*B*), and sidechain orientations of I223 (*C*) and L223 (*D*) residues.

ESM_2

A

Human KIR1.1	217	R	K	S	L	L	I	G	S	H	I	Y	227
Human KIR2.1	218	R	K	S	H	L	V	E	A	H	V	R	228
Human KIR2.2	219	R	K	S	H	I	V	E	A	H	V	R	229
Human KIR2.3	210	R	K	S	H	I	V	E	A	H	V	R	220
Human KIR2.4	223	R	R	S	H	L	V	E	A	H	V	R	233
Human KIR3.1	219	R	N	S	H	M	V	S	A	Q	I	R	229
Human KIR3.2	228	R	N	S	H	I	V	E	A	S	I	R	238
Human KIR3.3	196	R	S	S	H	I	V	E	A	S	I	R	206
Human KIR3.4	225	R	N	S	H	I	V	E	A	S	I	R	235
Human KIR4.1	204	R	K	S	L	L	I	G	C	Q	V	T	214
Human KIR4.2	203	R	K	S	L	L	I	Q	C	Q	L	S	213
Human KIR5.1	207	R	P	N	H	V	V	E	G	T	V	R	217
Human KIR6.1	216	R	K	S	M	I	I	S	A	S	V	R	226
Human KIR6.2	206	R	K	S	M	I	I	S	A	T	I	H	216
Human KIR7.1	195	R	P	S	P	L	T	S	V	R	V	S	205

B

Mouse KIR1.1	218	R	K	S	L	L	I	G	S	H	I	Y	228
Mouse KIR2.1	218	R	K	S	H	L	V	E	A	H	V	R	228
Mouse KIR2.2	219	R	K	S	H	I	V	E	A	H	V	R	229
Mouse KIR2.3	209	R	K	S	H	I	V	E	A	H	V	R	219
Mouse KIR2.4	223	R	R	S	H	L	V	E	A	H	V	R	233
Mouse KIR3.1	219	R	N	S	H	M	V	S	A	Q	I	R	229
Mouse KIR3.2	230	R	N	S	H	I	V	E	A	S	I	R	240
Mouse KIR3.3	196	R	S	S	H	I	V	E	A	S	I	R	206
Mouse KIR3.4	225	R	N	S	H	I	V	E	A	S	I	R	235
Mouse KIR4.1	204	R	K	S	L	L	I	G	C	Q	V	T	214
Mouse KIR4.2	230	R	K	S	L	L	I	Q	C	Q	L	S	240
Mouse KIR5.1	207	R	P	N	H	V	V	E	G	T	V	R	217
Mouse KIR6.1	216	R	K	S	M	I	I	S	A	S	V	R	226
Mouse KIR6.2	206	R	K	S	M	I	I	S	A	T	I	H	216
Mouse KIR7.1	195	R	P	S	P	L	T	N	V	R	V	S	205
Chicken Kir2.2	219	R	K	S	H	I	V	E	A	H	V	R	229

ESM_2: Sequence alignments of the CD loop for human (*A*) and mouse (*B*) Kir channels. The chicken Kir2.2 sequence was also aligned with the mouse Kir channels, and the residue that was focused in this study was marked in yellow.

ESM_3

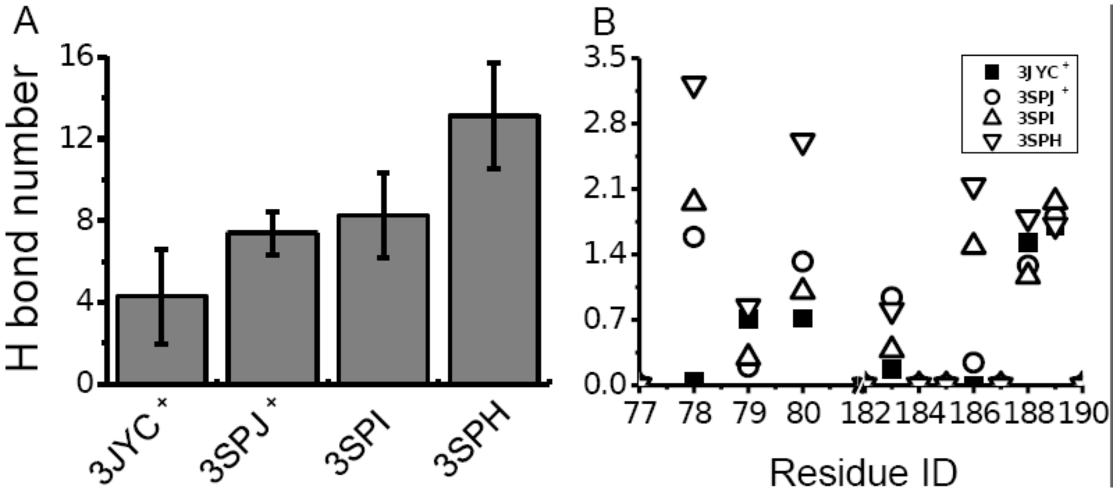

ESM_3: Global H bond interactions (*A*) and the distributions among binding sites (*B*) for PIP_2-liganded chicken Kir2.2 systems. The data was presented as mean ± SD (*A*) or mean only (*B*) of tetramer after the last 10-*ns* trajectory average of each subunit.

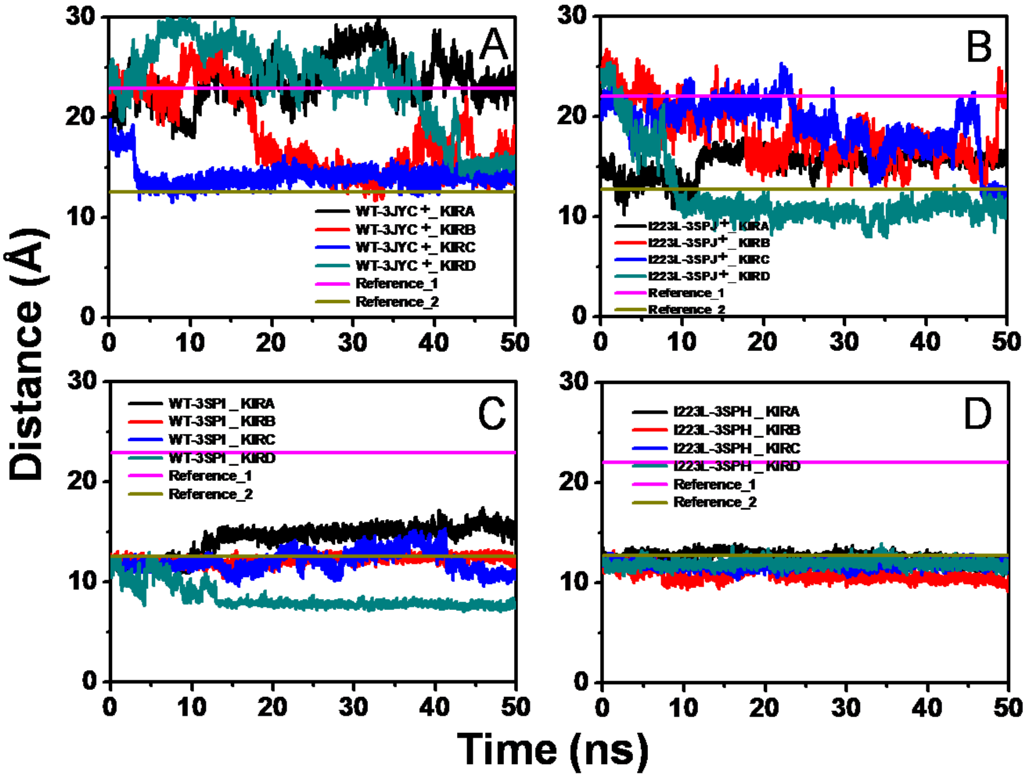

ESM_4: Evolution of R186-R78 distance for PIP_2-liganded chicken Kir2.2 systems. The evolution along 50-*ns* equilibration of each subunit was presented for artificially PIP_2-added WT (*A*) and I223L mutant (*B*) systems, as well as PIP_2 co-crystallized WT (*C*) and I223L mutant (*D*) systems. The references 1 and 2 were the corresponding R78-R186 distances of crystallized PIP_2-absent (reference_1) and PIP_2-presence (reference_2) WT and I223L mutant structures.

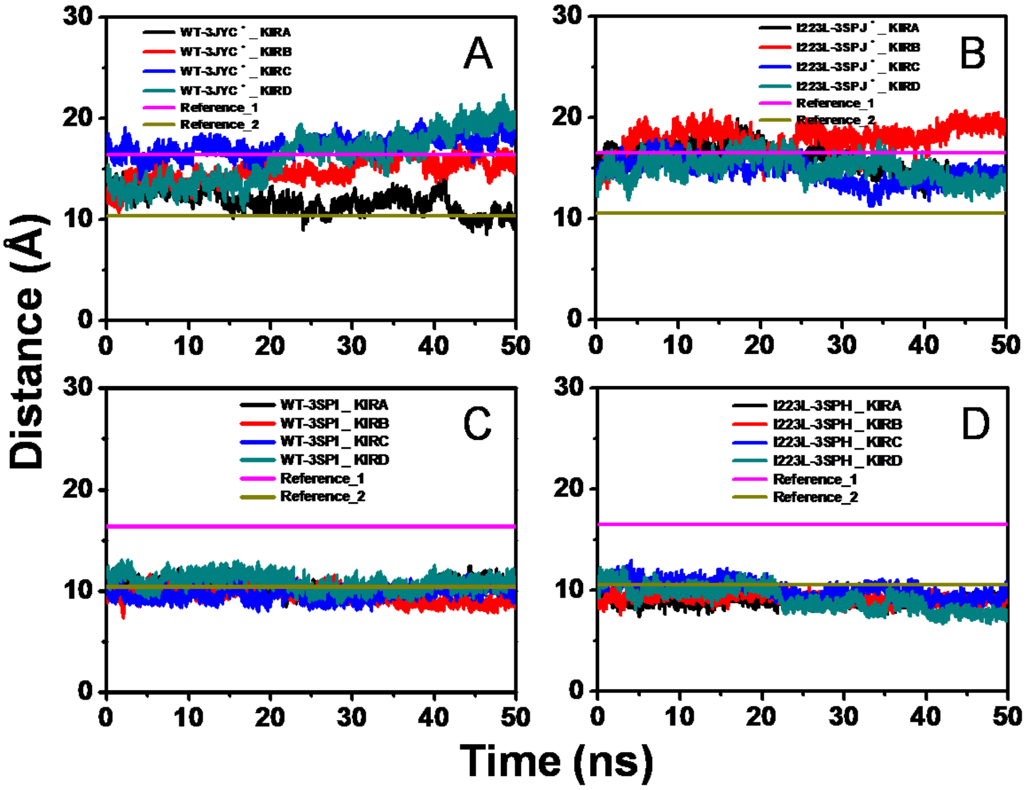

ESM_5: Evolution of TMD-CTD distance for PIP_2-liganded chicken Kir2.2 systems. The evolution along 50-*ns* equilibration of each subunit was presented for artificially PIP_2-added WT (*A*) and I223L mutant (*B*) systems, as well as PIP_2 co-crystallized WT (*C*) and I223L mutant (*D*) systems. The references 1 and 2 were the corresponding TMD-CTD distances of crystallized PIP_2-absent (reference_1) and PIP_2-presence (reference_2) WT and I223L mutant structures.

ESM_6

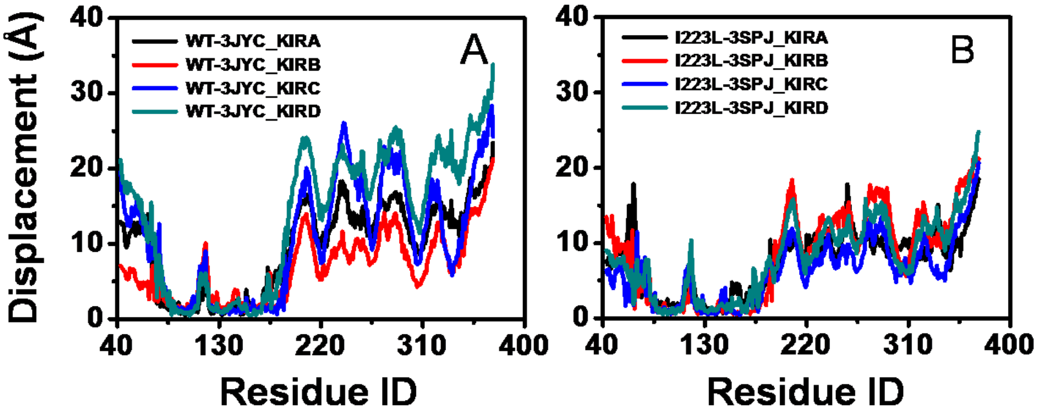

ESM_6: Conformational stability of PIP_2-absent chicken Kir2.2 WT (*A*) and I223L mutant (*B*) systems. The displacement for each residue was defined as the distance between the heavy atom center of equilibrated snapshot and that of the initial crystallized structure upon the alignment of the backbone atoms of transmembrane M1 (L85-A105) and M2 (C155-I180) helixes, and the data were shown as the average of last 10-*ns* equilibration.
